# Zinc ion increases the effectiveness of phosphorus in agricultural soils through microbial solubilization

**DOI:** 10.1371/journal.pone.0327961

**Published:** 2025-12-15

**Authors:** Lanxin Tang, Bingrui Su, Xuehao Zheng

**Affiliations:** 1 School of Geographical Sciences, China West Normal University, Nanchong, China; 2 School of Resource and Environmental Sciences, Wuhan University, Wuhan, China; Universidade de Coimbra, PORTUGAL

## Abstract

Phosphorus (P) is a key limiting factor for crop growth in agroecosystems, where soil microorganisms and mineral ions are essential for P turnover and mobilization. However, it is unclear how mineral ions affect microbially-mediated phosphorus cycling. In this study, 15 soil samples were collected from a typical agricultural area in Southwest China with a complex mineral composition. Soil phosphorus levels, microbial phosphorus genes, and typical soil mineral ions were subsequently analyzed to determine the mechanism of soil mineral ions on phosphorus cycling. The results showed that Zn^2+^ was the main driver of P mobilization in the soils, and its accumulation led to changes in the expression of two important phosphorus genes, *gcd* and *phnW*. Ecological clusters dominated by Actinobacteria and Proteobacteria, phyla that carry the *gcd* gene, directly contributed to P activation through mutualistic interactions. *phnW* encodes a phosphorylglycolide hydrolase enzyme involved in phosphate accumulation, and its transcription may enhance the soil available phosphorus (AP) content by promoting enzymatic carbon-phosphorus bond cleavage, a process influenced by microbes in the phyla Proteobacteria, Gemmatimonadetes, Actinobacteria, and Acidobacteria. Herein, our work provides a new perspective on the mechanism of P cycling and improve P effectiveness in agricultural soils.

## 1. Introduction

Phosphorus (P) is a key limiting factor for crop growth in agroecosystems, being involved in biological metabolism and energy conversion [1,2]. In terrestrial ecosystems, less than 6% of the P is bioavailable [3]. In addition, Available phosphorus (AP) concentrations in 21 representative U.S. agricultural soils exhibited values predominantly below 10μM, with inorganic phosphorus (Pi) concentrations averaging 3μM [4]—levels substantially below the threshold required for optimal crop growth. This can lead to significant P limitation in agricultural practice [5,6]. The situation is exacerbated by negative ecological effects of human activities such as atmospheric nitrogen deposition and soil carbon cycle imbalance, thus posing challenges to agricultural sustainability [3,7,8]. Improving the efficiency of P utilization and increasing the soil AP is a major priority for agroecosystem management and soil restoration.

Soil AP is influenced by various factors, such as mineral ions and microorganisms [9,10]. Microorganisms play a crucial role in ecosystems and are characterized by their generalized interactions [11,12]. Microorganisms can interact positively through the cooperation and negatively through indirect exploitative competition and direct interference competition [12]. However, most studies of the P cycle and AP have focused on individual microbe species [13], neglecting microbial community interactions in natural ecosystems. Given the diversity of microbial communities, their interactions may significantly impact the P cycle [14]. Therefore, the role of microbial interactions among specific groups in agricultural soils in response to P limitation and soil AP enhancement should be considered.

Mineral ions in the soil affect the soil P cycle and the level of AP. Various mineral ions can adsorb and precipitate soil AP. For example, Fe^3+^ and Al^3+^ can form phosphate minerals in acidic soils [15], while in alkaline or calcareous soils with a high Ca^2+^ content, phosphate ions readily combine with Ca^2+^ to form calcium phosphate precipitates [16], thereby reducing soil AP. Zn^2+^ is widely distributed in soils worldwide and readily combines with phosphate to form insoluble zinc phosphate [17]. While the majority of mineral ions diminish phosphorus bioavailability, select ions can paradoxically facilitate phosphorus mobilization through microbial mediation under particular circumstances. For example, Sb^2+^ increases soil phosphatase activity and alters the bacterial community structure, thereby enhancing soil AP [18]. Mg^2+^ has a significant effect on microbial metabolic activities; appropriate Mg^2+^ levels help maintain microbial community health and activity [19], indirectly influencing soil AP. Therefore, understanding the complex effects of mineral ions on P cycling is essential, particularly the mechanisms by which soil microbially-mediated mineral ions affect P cycling and soil AP in farmland ecosystems.

In summary, soil mineral ions and microbial activities play crucial roles in the soil P cycling process, but their interactions are not well understood. To investigate the mechanisms by which mineral ions affect microbially-mediated P activation in agroecosystems, we conducted mesoscale sampling of agricultural soils in the representative agricultural area in Sichuan Province, China. By comprehensively analyzing important mineral ions and microbial phosphorus-cycling genes in the soil, we hypothesize that (1) Zn^2+^ is the key mineral ion regulating soil AP; (2) Zn^2+^ regulates soil AP mainly by influencing the microbial phosphorus solubilization process; and (3) some of the specific microbial communities during phosphorus solubilization contribute to the activation of phosphorus by enhancing interactions. These findings will provide new insights for managing and improving P efficiency in agricultural soils and thereby offer a theoretical basis for mitigating soil P limitation.

## 2. Materials and methods

### 2.1. Soil sample collection

**Fig 1:**
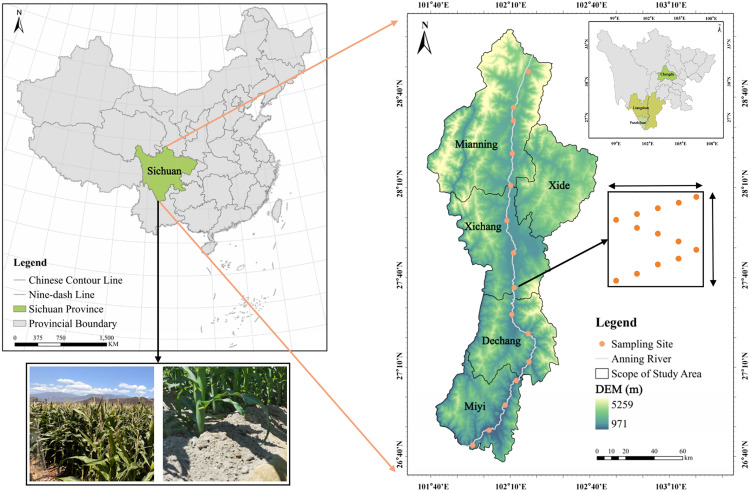
Locations of the sampling points. (A) The study area is situated in the southwestern part of Sichuan Province, China, and the 15 sampling sites cover Mianning, Xide, Xichang, and Dechang counties in Liangshan Yi Autonomous Prefecture and Miyi County in Panzhihua City. (B) The “S” sampling method was used for each sampling point.

The study area was located in the Anning River Basin (101.93°E-102.28°E, 26.73°N-28.81°N) (Fig 1) in the southwestern part of Sichuan Province, a major agricultural region in China. The basin encompasses 5.77 million hectares of arable land, with maize being the predominant crop. Situated at the eastern edge of the Qinghai-Tibetan Plateau, the region features a unique geological structure and soil environment, resulting in a rich mineral composition in the agricultural soils [20,21]. This complexity in P adsorption and resolution makes it an ideal area for studying the influence of soil mineral ions on the P cycle.

In addition to four basic sampling sites in the Anning River Basin established by the Ministry of Ecology and Environment of China, we set up 11 additional sampling sites, resulting in a total of 15 sites, each spaced approximately 20 km apart. At each sampling site, we selected 16 × 20 m plots of farmland that had been exclusively planted with maize for the past 10 years. All 15 sites were located in the plain area of the riparian zone within 100m ~ 200m from the Anning River. Among them, S1 ~ S5 and S9 are red soil, and S6 ~ S8 and S10 ~ S15 are yellow soil. The soil pH ranges from about 5.8–9.3. The maize planting variety in each sample point is “Chuan Dan 99”, and all of them apply about 15 kg of NPK compound fertilizer (P fertilizer content of 10% ~ 12%) per mu during the period of maize pulling out, and do not apply fertilizer during the period of tasseling. Therefore, we chose to sample the plots during the tasseling period to ensure that there was no obvious residual fertilizer.

Using a sterile shovel, we collected soil from 13 sample points arranged in an “S” shape within each plot, gathering surface soil from a depth of 0–20 cm. At each “S”-shaped point, we collected 100g of soil, resulting in a total of 1300g of soil collected from each plot. Soil impurities were removed under aseptic conditions, and the 13 samples from each plot were combined to create a composite sample representative of the plot. These composite samples were stored in sterile Ziploc bags, kept on dry ice, and immediately transported to the laboratory. Each composite sample was then divided into three subsamples: one for microbiological testing (50g was used for this purpose), one that was air-dried and ground through a 60-mesh sieve for soil characterization, and one retained as a backup.

### 2.2. Analysis of soil properties

Soil total phosphorus (TP) was determined by sulfuric acid and perchloric acid digestion [14]. Soil AP was extracted with 0.5 M sodium bicarbonate and then analyzed using the ammonium molybdate-ascorbic acid method, with the absorbance at 700 nm measured using a microplate reader (Varioskan LUX, Thermo Scientific, USA) [18]. Soil Pi was determined by the “The Standards, Measurements and Testing Programme” method, while soil organic phosphorus (Po) was calculated as the difference between TP and Pi [18]. Soil mineral ions (Ca^2+^, Mg^2+^, Al^3+^, Zn^2+^, Mn^2+^, Cu^2+^) and water-soluble Fe (WS-Fe) were determined by inductively coupled plasma mass spectrometry (ICP-MS: Agilent 7800, USA). Water-soluble Si (WS-Si) was determined by inductively coupled plasma emission spectrometry (ICP-OES: Agilent 5110, USA).

### 2.3. DNA extraction, library construction, and metagenomic sequencing

The total genomic DNA was extracted from 15 samples using the Mag-Bind® Soil DNA Kit (Omega Bio-tek, Norcross, GA, USA) according to manufacturer’s instructions. The concentration and purity of extracted DNA were determined using the TBS-380 fluorometer (Turner BioSystems, USA) and NanoDrop2000 (Thermo Fisher Scientific, Massachusetts, USA) respectively. The DNA extract quality was checked on 1% agarose gel.

The DNA extract was fragmented to an average size of about 400 bp using the Covaris M220 (Gene Company Limited, China) for paired-end library construction. The paired-end library was assembled using NEXTFLEX Rapid DNA-Seq (Bioo Scientific, Austin, TX, USA). Adapters containing the full complement of sequencing primer hybridization sites were ligated to the blunt ends of fragments. Paired-end sequencing was performed on the Illumina NovaSeq (Illumina Inc., San Diego, CA, USA) at Majorbio Bio-Pharm Technology Co., Ltd. (Shanghai, China) using the NovaSeq 6000 S4 Reagent Kit v1.5 (300 cycles) according to the manufacturer’s instructions (www.illumina.com). Sequence data associated with this project have been deposited in the National Center for Biotechnology Information (NCBI) Short Read Archive database (Accession Number: PRJNA1223346).

### 2.4. Gene prediction, taxonomy, and functional annotation

The data were analyzed on the free online Majorbio Cloud Platform (www.majorbio.com). Briefly, the paired-end Illumina reads were trimmed to remove adaptors, and low-quality reads (reads with a length<50 bp, a quality value <20, or N bases) were removed using fastp [22]. Metagenomic data were assembled using MEGAHIT [23], which makes use of succinct de Bruijn graphs. Contigs with a length ≥ 300 bp were selected as the final assembly result, and the contigs were employed for further gene prediction and annotation.

Open reading frames (ORFs) from each assembled contig were predicted using Prodigal/MetaGene [24,25]. The predicted ORFs with a length ≥ 100 bp were retrieved and translated into amino acid sequences using the NCBI translation table. A non-redundant gene catalog was constructed using CD-HIT with 90% sequence identity and 90% coverage [26]. High-quality reads were aligned to the non-redundant gene catalogs to calculate the gene abundance with 95% identity using SOAPaligner [27]. Representative sequences from the non-redundant gene catalog were aligned to the non-redundant (NR) database with an e-value cutoff of 1e^−5^ using Diamond for taxonomic annotations [28]. Clusters of Orthologous Groups of proteins (COG) annotation for the representative sequences was performed using Diamond against the eggNOG database with an e-value cutoff of 1e^−5^. Kyoto Encyclopedia of Genes and Genomes (KEGG) annotation was conducted using Diamond against the KEGG database with an e-value cutoff of 1e^−5^.

### 2.5. Statistical analysis

The Shapiro-Wilk test and Levene’s test were used to assess normality and the homogeneity of variance, respectively. Experimental data are expressed as mean ± standard deviation of three replications. Data analysis was performed in R (v4.3.3). Spearman correlation analysis was used to assess the relationship between parameters. Heatmaps illustrating the abundance of P-cycle genes in the metagenomic data were constructed using the “pheatmap” R package [29]. Based on known and potential relationships, a partial least squares path model (PLS-PM) was constructed using the “plsmp” R package to explore the effects of biotic and abiotic factors (e.g., phosphorus-cycling genes, mineral ions, and TP) on soil AP, with variable selection based on correlation analysis. The goodness-of-fit (GoF) index was used to assess the overall fit of the model [30], being categorized as weak (GoF > 0.1), moderate (GoF > 0.25), or strong (GoF > 0.36) [31]. Random forest (RF) analyses were performed using the “randomForest” package to identify microbial P-cycling genes that affected soil AP, with significance evaluated by 1000 permutations using the “rfPermute” and “A3” R packages, respectively [32]. The “igraph” R package was used to construct a network of key soil microbial communities at the species level to determine whether microbial-mediated P cycling was influenced by mineral ions and how it affected the complexity of ecological networks [33]. P values were adjusted using the Benjamini and Hochberg false discovery rate (FDR) test, with a cut-off value of 0.001 [34]. The network topology of soil samples was visualized using the interactive platform “Gephi.”

## 3. Results

### 3.1. Soil P fractions and mineral ion content

**Fig 2:**
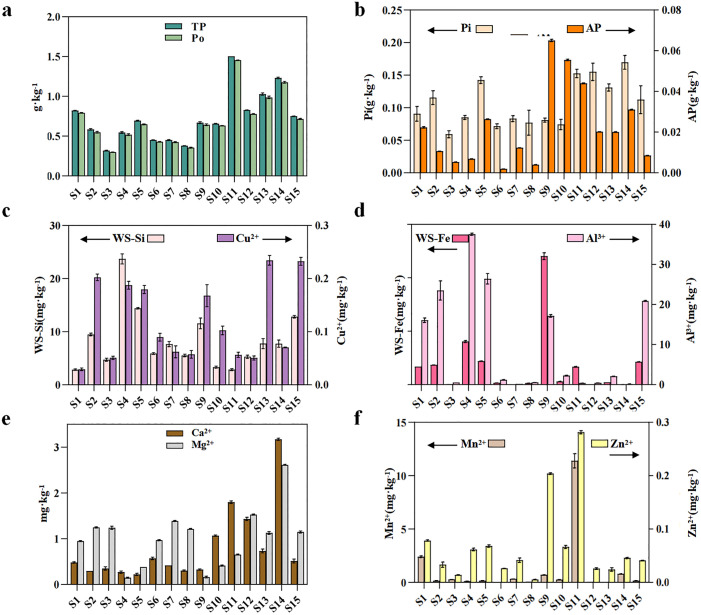
Soil phosphorus contents and mineral ion contents. (A) Total and organic phosphorus contents. (B) Inorganic and available phosphorus contents. (C) Silicon and copper ion contents. (D) Iron and aluminum ion contents. (E) Calcium and magnesium ion contents. (F) Zinc and manganese ion contents. TP: Total phosphorus, Po: Organic phosphorus, Pi: Inorganic phosphorus, AP: Available phosphorus.

The soil TP ranged from 0.31 to 1.50 g·kg^-1^ (Fig 2a); the Po ranged from 0.30 to 1.46 g·kg^-1^(Fig 2a); the Pi ranged from 0.02 to 0.06 g·kg^-1^ (Fig 2b), and the AP ranged from 0.01 to 0.21 g·kg^-1^(Fig 2b). These data were compared to the P contents of other typical farmland soils in China (S1 Table, S1 Fig). The TP in the farmland soils of the Anning River Basin was moderate, but the AP was highly variable. The AP content at S6 is extremely low, at just 0.01 g·kg^-1^, which is well below the commonly recognized threshold for phosphorus deficiency (e.g., 5−10 mg/kg, Fig 2b). Furthermore, the AP contents at S2, S3, S4, S8, S12, S13, and S15 are also relatively low, not exceeding 0.02 g·kg^-1^ (Fig 2b), indicating stronger P limitation in some areas.

Among the soil mineral ions, WS-Si ranged from 2.63 to 24.09 mg·kg^-1^; Cu^2+^ ranged from 0.03 to 0.25 mg·kg^-1^ (Fig 2c); WS-Fe ranged from 0.02 to 5.67 mg·kg^-1^; Al^3+^ ranged from 0.03 to 33.01 mg·kg^-1^ (Fig 2d); Ca^2+^ ranged from 0.20 to 3.20 g·kg^-1^; Mg^2+^ ranged from 0.14 to 2.62 mg·kg^-1^ (Fig 2e); Zn^2+^ ranged from 0.01 to 0.28 mg·kg^-1^, and the Mn^2+^ ranged from 0.02 to 11.79 mg·kg^-1^ (Fig 2f). There were significant differences among the sample points, with extremely low Ca^2+^ and Mg^2+^ contents at S4 and S9 and the highest content at S14. The Zn^2+^ and Mn^2+^ contents were extremely high at S11. The highest WS-Si content was at S4, while Cu^2+^ contents were high at S14 and S15. The WS-Fe content was extremely high at S9, and the Al^3+^ content was extremely high at S4.

### 3.2. The relationship between soil mineral ions and phosphorus fractions

**Fig 3:**
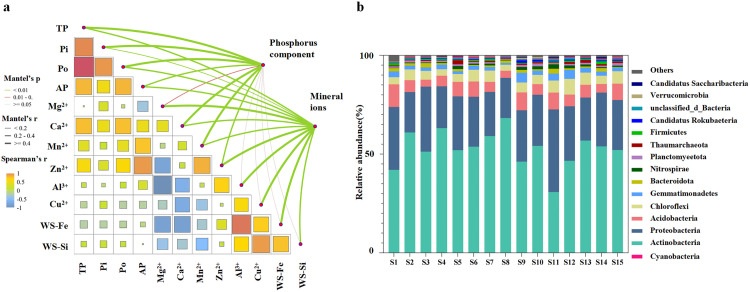
Correlations between soil P-vein fractions and mineral ions. (A) Correlations between soil P fractions and mineral ions. Edge width and color correspond to Mantel’s R and P values, respectively. (B) Pairwise Spearman correlations for soil variables indicated by color gradients. TP total phosphorus, Pi: inorganic phosphorus, Po: organic phosphorus, AP: available phosphorus, WS- Fe: water-soluble Fe, WS-Si: water-soluble Si. (B) Relative soil microbial abundance at the phylum level.

We examined the correlations between soil mineral ions and P. A Mantel analysis revealed that Ca^2+^, Mn^2+^, and Zn^2+^ showed strong correlations (*P *< 0.01) with P-containing components (Fig 3a). Spearman correlation analysis (S2 Fig) further clarified these relationships, indicating that various forms of P were correlated with different mineral ions. Specifically, AP showed significant positive correlations with Zn^2+^ and Mn^2+^ (*P *< 0.001) and with Ca^2+^ (*P *< 0.05). TP was significantly positively correlated with Ca^2+^ (*P *< 0.001) and Zn^2+^(*P *< 0.01). Pi was significantly positively correlated with Ca^2+^ (*P *< 0.05). Po was significantly positively correlated with Ca^2+^ and Zn^2+^ (*P *< 0.01) and Mn^2+^ (*P *< 0.01). These results demonstrate that many mineral ions were associated with AP in the Anning River Basin, with Zn^2+^ showing the strongest correlation.

### 3.3. Microbial community structure and phosphorus cycling gene abundance in the Anning River Watershed

Actinobacteria was dominant at the phylum level (Fig 3b). Actinobacteria, Proteobacteria, and Acidobacteria collectively comprised about 80% of the microbial community in the soil of the riparian zone of the Anning River. Other significant phyla included Chloroflexi, Gemmatimonadetes, Bacteroidota, Planctomycetota, Nitrospirae, Thaumarchaeota, and Firmicutes. S11 had significant differences in the abundances of microbial phyla compared to the other sites, with Proteobacteria being dominant at 41.8% (Fig 3b). This is in contrast to the other sites, where Actinobacteria was the dominant phylum.

**Fig 4:**
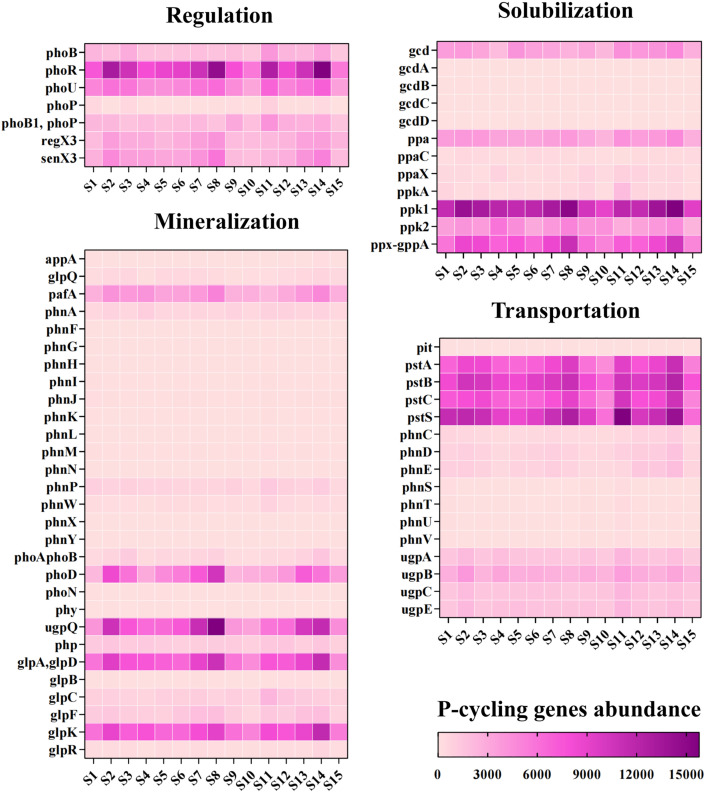
Relative abundance of soil phosphorus cycling genes. The genes are categorized as Phosphorus limitation regulatory function genes, Mineralization of organic phosphorus genes, Transportation function genes, and Solubilization of phosphorus genes.

A total of 64 P cycle-related genes were detected, along with their corresponding enzymes/proteins and KEGG IDs from the KEGG database (S2 Table). These genes were categorized into four P-transformation processes: mineralization of Po, solubilization of Pi, transportation, and regulation (Fig 4). Genes involved in P starvation regulation, such as *phoR* and *phoU*, were highly abundant. Genes associated with the mineralization of Po, such as *phoD*, *ugpQ*, *glpAD*, and *glpK*, also showed high abundance. Additionally, transporter function genes, including *pstABCS* and P solubilization genes such as *ppk12* and *gcd*, were highly abundant. The abundance of P cycling-related genes was significantly higher at S8 and S14 compared to the other sites.

### 3.4. Relationships between soil AP and soil mineral ions, microorganisms

**Fig 5:**
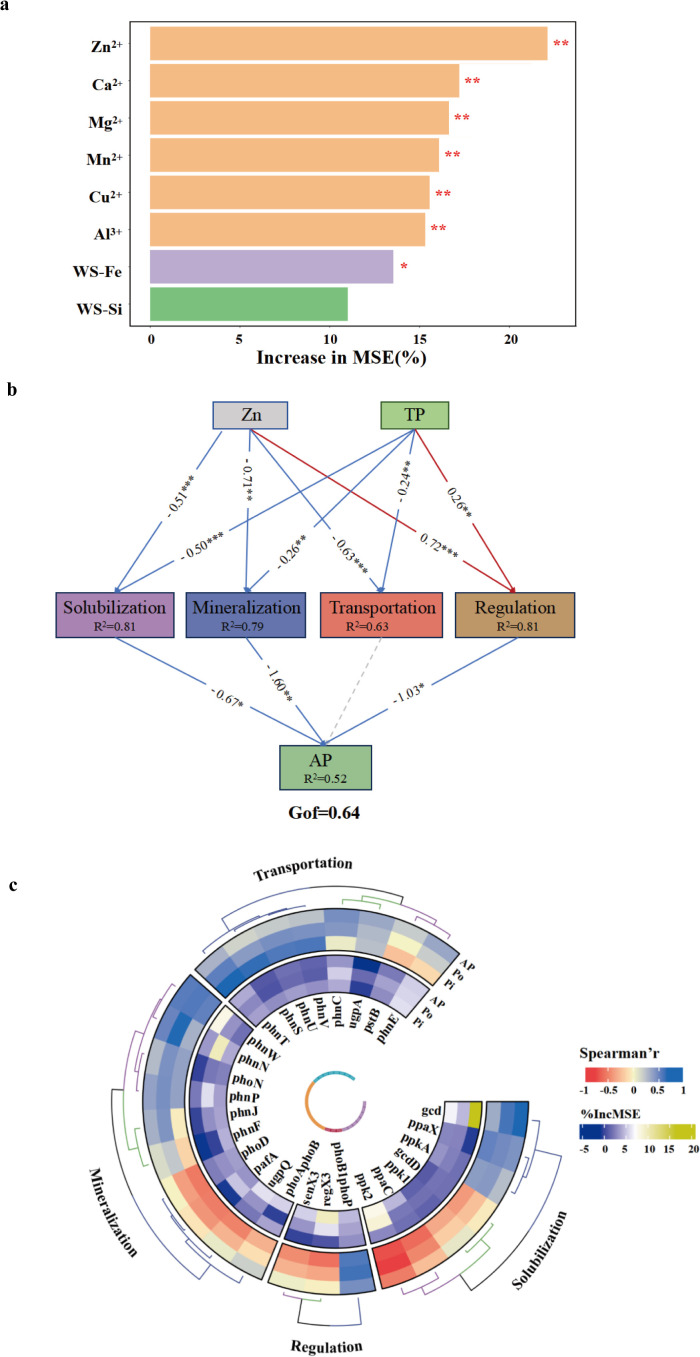
(A) Identification of dominant soil mineral ions acting on AP based on the Random Forest model. Orange (or **) represents *P* < 0.01, while purple (or *) represents *P* < 0.05. (B) Linkage between soil microbial phosphorus cycling genes and AP. PLS-PM analysis showed the effect of Zn^2+^ on the effectiveness of soil microbial utilization of phosphorus. Path coefficients, significance levels, and coefficients of determination (*R*^*2*^) were calculated using 1000 bootstrap replicates. The red and blue arrows represent positive and negative effects, respectively. (C) Identification of microbial phosphorus cycling genes based on Spearman correlation analysis and Random Forest modeling. The outer circle represents the Spearman correlation coefficients, with blue color indicating positive correlations and red color indicating negative correlations. The inner circle represents the predicted significance of Random Forest, and the outer bifurcation tree represents the genomic groups with strong correlations.

RF analysis was used to evaluate the key mineral ions predicted to influence soil AP. The results indicated that Zn^2+^ was the most important determinant of soil AP (*P* < 0.01, Fig 5a). Further statistical analysis using PLS-PM explained 52% of the total variation in soil microbial effectiveness in utilizing P (Fig 5b). Genes involved in P solubilization had a significant negative effect on soil AP (*P* < 0.05), while genes associated with P mineralization had a highly significant negative effect on soil AP (*P* < 0.01), and genes associated with P limitation regulation also had a significant negative effect on soil AP (*P *< 0.05).

Additionally, the mineral ions Zn^2+^ and TP affected soil AP by influencing microbial P cycling genes (Fig 5b). Specifically, Zn^2+^ had a highly significant negative effect on P solubilization genes (*R²* = 0.81, *P* < 0.001), and TP also had a highly significant negative effect on P solubilization genes (*P* < 0.001). Zn^2+^ similarly had a highly significant negative effect on P mineralization genes (*R²* = 0.79, *P* < 0.01), while TP had a highly significant negative effect on P mineralization genes (*P* < 0.01). In contrast, Zn^2+^ had a highly significant positive effect on P limitation regulation genes (*R²* = 0.81, *P* < 0.001). Thus, the pathway by which Zn^2+^ accumulation increased AP involved the downregulation of P solubilization and mineralization genes during microbial P cycling, which in turn increased soil AP.

The activity of solubilization genes was one of the most relevant factors for soil P bioavailability under the influence of Zn^2+^. Based on the RF analysis and Spearman correlation analysis, *gcd*, responsible for encoding the quinoprotein glucose dehydrogenase, was identified as the key gene determining the effectiveness of microbial P utilization among the solubilization genes (Fig 5c). The *gcd* gene was significantly positively correlated with soil AP compared to other genes (*P* < 0.05); its action involves directly controlling glucose oxidation pathways and acidification of the periplasmic interstitial space. These results suggest that P solubilization driven by *gcd*-carrying microorganisms under the influence of Zn^2+^ may be the primary process enhancing P bioavailability in farmland soils in the Anning River Basin.

### 3.5. Microbial co-occurrence networks containing the Solubilization gene *gcd*

**Fig 6:**
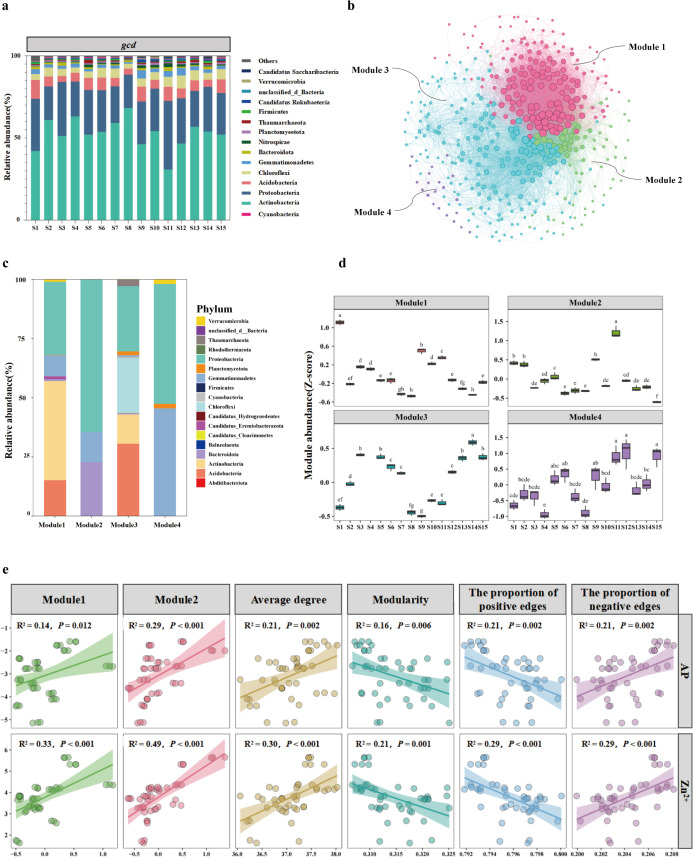
(A) Microbial composition analysis reveals key gates of phosphorus solubilizing microorganisms in agricultural fields of the Anning River Basin. (B) Clusters of phyla carrying the gcd soil microbial species-level co-occurrence network, with the network diagram displaying nodes colored according to each of the four main ecological clusters (Modules 1-4). (C) Quantitative characteristics of the dominant species in the major ecological clusters at the phylum level. (D) Abundance of the major ecological clusters in soils of the Annin River Basin. (E) Topology of the key ecological clusters and soil microbiota co-occurrence network with AP and soil Zn^2+^. The linear relationships between the topological features of the co-occurrence network of major ecological clusters and soil microbiota with AP and Zn^2+^ were analyzed using ordinary least squares linear regression. The topological features of the co-occurrence network included modularity, average degree, the proportion of positive edges, and the proportion of negative edges.

To further characterize the *gcd*-carrying microorganisms, we found that 30% to 60% of these bacteria were assigned to the Actinobacteria, followed by the Proteobacteria (21%–41%) and the Acidobacteria (3%–11%) across different sites (Fig 6a). The *gcd*-carrying bacteria in the entire study area were dominated by Actinobacteria and Proteobacteria, followed by Bacteroidota and Acidobacteria, with Alphaproteobacteria being the most numerous within the Proteobacteria (S3a Fig).

We then constructed a co-occurrence network of all *gcd*-carrying species, including those constituting more than 80% of the species in the network, and extracted sub-networks to identify the main ecological clusters at the level of four phyla (Modules1–4, Fig 6b). The relative abundance of each ecological cluster was calculated by averaging the standardized relative abundance (Z-score) of the species within each cluster. Module 1 contained the phyla Actinobacteria, Proteobacteria, Acidobacteria, Gemmatimonadetes, and Verrucomicrobia, with Actinobacteria and Proteobacteria being dominant. Module 2 comprised Proteobacteria, Bacteroidota, and Gemmatimonadetes, and was dominated by Proteobacteria. Module 3 was dominated by Acidobacteria, Actinobacteria, and Proteobacteria, and also included Chloroflexi, Cyanobacteria, Planctomycetota, and Bacteroidota. Module 4 was dominated by Proteobacteria and Gemmatimonadetes (Fig 6c). Module 1 had the highest relative abundance at S1; Module 2 was dominant at S11, and both Module 3 and Module 4 were the highest at S14 (Fig 6d).

To further explore the role of the four major ecological clusters carrying *gcd* in utilizing AP under the influence of Zn^2+^, we analyzed their relative abundance in relation to AP and Zn^2+^ (Fig 6e). The relative abundance of Module 2 was significantly positively correlated with AP and Zn^2+^ (*P* < 0.001). Module 1 showed significant positive correlations with AP (*P* < 0.05) and Zn^2+^ (*P* < 0.001). Thus, Module 2, dominated by Proteobacteria, and Module 1, dominated by Actinobacteria and Proteobacteria, were identified as the key ecological clusters enhancing P effectiveness through solubilization processes in agricultural soils of the Anning River Basin.

The topological features of the co-occurrence network of soil microbiota were further analyzed for their linear relationships with AP and Zn^2+^ (Fig 6e). The average degree showed significant positive correlations with AP (*R²* = 0.21, *P* < 0.01) and Zn^2+^ (*R²* = 0.23, *P* < 0.001). Modularity was significantly negatively correlated with AP (*R²* = 0.16, *P* < 0.01) and negatively correlated with Zn^2+^ (*R²* = 0.19, *P* < 0.01). The proportion of positive edges was significantly negatively correlated with AP (*R²* = 0.21, *P* < 0.01) and negatively correlated with Zn^2+^ (*R²* = 0.25, *P* < 0.001). The proportion of negative edges was positively correlated with AP (*R²* = 0.21, *P* < 0.01) and Zn^2+^ (*R²* = 0.25, *P* < 0.001). The average degree reflected the interaction strength of microorganisms in the network, indicating that during the *gcd* gene-mediated P solubilization process, microbes further enhanced soil P effectiveness by increasing interaction strength. Stronger microbial interactions were correlated with higher soil AP.

### 3.6. Correlation analysis of the abundance of genes in key pathways of the Zn^2+^ and phosphorus cycles

**Fig 7:**
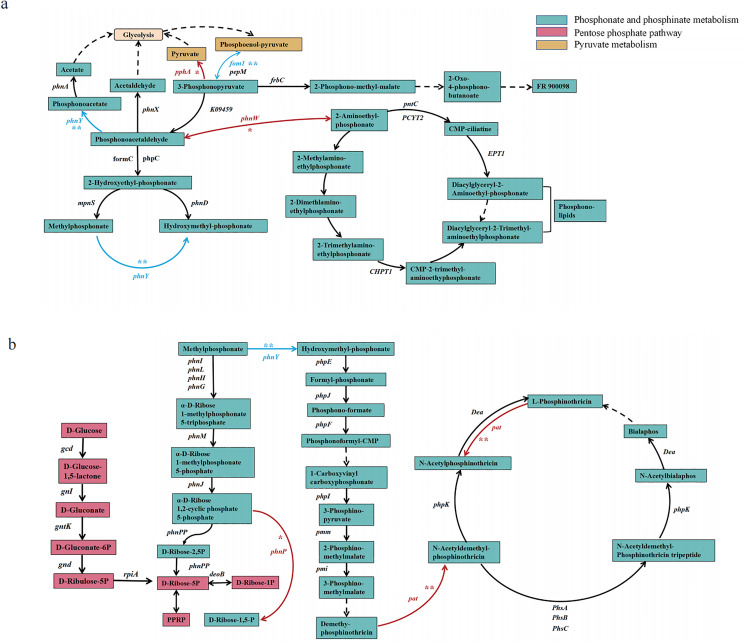
Typical pathways of phosphorus cycling influenced by Zn^2^^+^, including phosphonate and phosphinate metabolism, the pentose phosphate pathway, and pyruvate metabolism.

In detail, in pyruvate metabolism, soil Zn^2+^ accumulation exhibited a significant negative correlation with the abundance of *fom1* (Fig 7a), which encodes a transcriptional activator involved in cell proliferation. Conversely, in phosphonate and phosphinate metabolism pathways, soil Zn^2+^ demonstrated significant positive correlations with the abundance of inorganic phosphatase genes including *pphA*, *phnW* (encoding 2-aminoethylphosphonate-pyruvate transaminase), *phnP* (encoding phosphoribosyl 1,2-cyclic phosphate phosphodiesterase), and *pat* (encoding phosphinothricin acetyltransferase) (Fig 7a). Notably, Zn^2+^ also showed a significant negative correlation with *phnY* abundance (encoding acetoacetaldehyde oxidase), thereby influencing the hydrolytic degradation of aminoethylphosphonate (AEP) within the P-cycle phosphonate and phosphinate metabolic pathways. Furthermore, a significant positive correlation was observed between Zn^2+^ concentration and *phnP* abundance during the C-P lyase-mediated breakdown of phosphonate and hypophosphate compounds in the P cycle (Fig 7b).

## 4. Discussion

### 4.1. Zn^2+^ accumulation promotes ecological interactions of microorganisms carrying *gcd* gene and thereby enhances AP

Our results systematically address the hypotheses on Zn^2^ ⁺ -microbial-AP linkages: the ecological clusters dominated by Actinobacteria and Proteobacteria are key to enhancing AP through P solubilization under the influence of Zn^2+^ in the agricultural soils of the Anning River Basin.

In response to P limitation, soil microorganisms re-established potential associations and form functional clusters to ameliorate nutrient limitation. Actinobacteria, the major microbial phylum in the soil of the Anning River Basin, is crucial for solubilizing Pi under the influence of Zn^2+^, thereby increasing AP. Actinobacteria had the highest percentage of *gcd*-carrying bacteria (Fig 6a), and many strains have been reported as phosphate-solubilizing bacteria (PSB) [35]. Strains of the genus *Streptomyces* within Actinobacteria are associated with crop growth and health [36]. Therefore, in the agricultural ecosystems of the Anning River Basin, Actinobacteria remains essential for solubilizing Pi.

Importantly, this study identified Module 2, a Proteobacteria-dominated ecological cluster that included species carrying *gcd* genes under the influence of Zn^2+^, and Module 1 dominated by Actinobacteria and Proteobacteria, as key ecological clusters for AP enhancement through Pi solubilization in the agricultural soils of the Anning River Basin (Fig 6e). Their interactions jointly mediated AP accumulation in the soil (Fig 6e). Ecological clusters represent important units and provide the opportunity to identify highly connected taxa [37,38]. The network analysis indicated that the ecological clusters dominated by Actinobacteria and Proteobacteria were key to enhancing AP through Pi solubilization, with their interactions being a critical factor (Figs 6a, 6e). Linear regression analysis showed that the average degree, reflecting the interaction strength of microorganisms in the network, was significantly positively correlated with AP and Zn^2+^ (Fig 6e). This suggests that microorganisms in these clusters enhance soil AP by increasing the strength of their interactions during P solubilization under the influence of Zn^2+^. The proportion of positive edges generally represents cooperative relationships such as cross-feeding, mutualistic symbiosis, and commensalism, interactions that are facilitated by common barriers to dispersal and similar environmental needs [39]. Conversely, the proportion of negative edges may indicate competition among microorganisms due to limited resources, ecological niches, and spatial isolation [40]. The positive correlations between negative edges, Zn^2+^, and AP (Fig 6e) suggest that increased competition among microorganisms under Zn^2+^ influence promotes the efficiency of P utilization. Therefore, Zn^2+^ enhances AP by influencing microbial interrelationships through two possible mechanisms: (1) increasing the intensity of microbial interactions during P solubilization and (2) increasing the level of competition among microorganisms.

The reasons why Zn^2+^ enhances AP by improving microbial interaction strength and competition are mainly as follows: (1) Zn^2+^ can serve as a cofactor for numerous enzymes (currently known to exceed 300 types) within microorganisms, stabilizing the folded conformation of metalloproteins and participating in vital intracellular biochemical reactions at a global level [41]. Specifically, it can act as a catalytic cofactor for alkaline phosphatase and alcohol dehydrogenase in the phosphorus cycle [42,43]. When Zn^2+^ supply in soil is sufficient, enzyme activity increases, thereby enhancing microbial metabolic activities and elevating the growth rates and accumulation of metabolites in these microorganisms. On one hand, these enzymes directly facilitate the activation of phosphorus to generate AP; on the other hand, these metabolites can serve as a nutrient source for other microorganisms, indirectly promoting interactions among phosphorus-solubilizing microorganisms to produce AP. (2) Quorum Sensing (QS) is a system that regulates gene expression and alters bacterial and microbial community behavior by perceiving the concentration threshold of specific quorum sensing signal molecules (QSSM) [44]. The QS process can modify the patterns of microbial interactions, namely cooperation and competition [45]. Zn^2+^ and zinc transport proteins can act as signaling mediators [46], thereby influencing the metabolic pathways and gene expression of phosphorus-solubilizing microorganisms to regulate their interactions (e.g., enhancing interaction strength) and increase AP. (3) Zinc can alter the dominant species in the microbial community, with these microorganisms gaining a competitive advantage under high zinc conditions by producing zinc-binding molecular products The zinc-induced competitive pressure drives community restructuring through dual selection: (a) Structural dominance: Actinobacteria exhibit zinc resistance via Zur proteins, outcompeting zinc-sensitive strains; *Streptomyces coelicolor* (a model Actinobacteria) has been found to possess zinc-binding Zur that activates zinc-dependent genes [47,48], and Actinobacteria has been proven to be one of the key bacteria for activating inorganic phosphorus [49]. This suggests that under high zinc conditions, Actinobacteria, as a dominant species, can promote inorganic phosphorus activation and increase AP, which is consistent with our results. (b) Functional specialization: Proteobacteria develop complementary strategies like biofilm-mediated spatial organization [50,51], optimizing P-solubilization efficiency.

These results confirm the three hypotheses: (1) Zn^2^ ⁺ is the key regulator of AP; (2) primarily through microbial P-solubilization processes; (3) mediated by Actinobacteria-Proteobacteria interaction networks that enhance functional complementarity and competitive optimization. These interactions are crucial to the diversity and functionality of soil microbial communities in the Anning River Basin.

### 4.2. Zn^2+^ promotes microbially-mediated AP accumulation by affecting three important pathways

The metabolic pathways associated with the P cycle include (1) phosphonate and phosphinate metabolism, (2) the pentose phosphate pathway, and (3) pyruvate metabolism. The metabolic pathways targeted by Zn^2+^ in this study are strategically positioned at the nexus of microbial P acquisition and central carbon metabolism, providing a mechanistic basis for their preferential regulation [52]. The conventional view is that Zn^2+^ can form zinc phosphate precipitates with P, thereby reducing the effectiveness of P. However, this study found that an increase in the Zn^2+^ content within a certain range may enhance P mobilization, i.e., the AP content (Fig 5b), through the modulation of microbial activity.

To clarify the effect of microorganisms carrying these related genes on AP under the influence of Zn^2+^, the network analysis revealed that the abundance of ecological clusters dominated by Proteobacteria, Gemmatimonadetes, Actinobacteria, and Acidobacteria under Zn^2+^ influence had highly significant positive correlations with AP (S3a, S3b Figs, S5 Fig). This evidence of Zn^2+^ influencing P cycle pathways and microbial interactions to enhance AP highlights the complexity of mineral ion and microbial interactions in the P cycle. Therefore, Zn^2+^ is more likely to act as a nutrient involved in microbial life activities, which in turn affect the P cycle, rather than as a chemical influencing P mineralization, and as a nutrient in the microbial P cycle, Zn^2+^ may be involved in the following two pathways.

First, Zn^2+^ accumulation significantly affected the AEP hydrolytic degradation pathway for phosphate generation from P cycle phosphonate and hypophosphite metabolism in this study. Microorganisms under the influence of Zn^2+^ preferentially generated phosphate via the hydrolytic pathway of *phnW*-encoded phosphoacetaldehyde hydrolase in the P cycle (Fig 7a) to enhance AP. AEP hydrolytic degradation consists of two main pathways: one pathway starts from the conversion reaction of AEP to phosphoacetaldehyde (PAA) catalyzed by *phnW*-encoded aminotransferases, and then PAA undergoes hydrolysis by *phnX*-encoded phosphoacetaldehyde hydrolase to generate acetaldehyde and inorganic phosphates [53]; in the other pathway, PAA is first converted to phosphoacetate by a *phnY*-encoded dehydrogenase and is then hydrolyzed by a *phnA*-encoded enzyme to generate acetate and phosphate [54]. Zn^2+^ accumulation significantly upregulated the abundance of *phnW* but down-regulated the abundance of *phnY*. This suggests that microorganisms under the influence of Zn^2+^ preferentially generate phosphate via the P cycle phosphorylglyoxal hydrolase hydrolysis pathway. Combined with the phosphonate and phosphinate metabolism in which *phnW* and *phnY* function (Fig 7a), we hypothesized that Zn^2+^ may affect the AEP hydrolysis degradation pathway in several ways. First, Zn^2+^ may act by increasing the tolerance to Zn^2+^ through adjusting relevant gene expression. Microorganisms can adapt to the concentrations of metal ions in the environment by regulating gene expression [55], and microorganisms may increase *phnW* expression and decrease *phnY* expression to adapt to the elevated Zn^2+^ concentration in the environment. Second, Zn^2+^ may act through the activities of related transcription factors. Zn^2+^ controls intracellular events by regulating some Zn-dependent proteins, including transcription factors [56]. These transcription factors’ activities are modulated by Zn^2+^ concentrations. Zn^2+^ could bind to specific metal-binding sites on these transcription factors, altering their DNA-binding affinity or transcriptional activity [57,58]. For instance, Zn^2+^ binding to a transcription factor might enhance its ability to bind to specific promoter regions of genes involved in the AEP hydrolytic degradation pathway, such as *phnW*, thereby upregulating its expression. Conversely, Zn^2+^ binding could also interfere with the binding of transcription factors to *phnY* promoters, leading to its downregulation. Furthermore, the Zn^2+^-mediated regulation of transcription factors also involve interactions with other signaling molecules and regulatory proteins, forming a complex regulatory network [59,60]. Zn^2+^ might act synergistically or competitively with other metal ions or small molecules to modulate the activity of these transcription factors, further refining the microbial response to Zn^2+^ concentration variations.

Zn^2+^ accumulation significantly promoted *phnP* in the C-P cleaving enzyme process in phosphonate and hypophosphite metabolism in the P cycle. We hypothesized that Zn^2+^ may promote this process by the following: (1) preferentially binding inorganic salt ions to form precipitates to release mineral ions and inhibiting the crop uptake of mineral ions, where the enzyme encoded by *phnP* was predicted by sequence homology to be a metal-dependent hydrolase of the β-lactamase superfamily, with a clear catalytic preference for Ni^2+^, suggesting that Zn^2+^ may preferentially bind to inorganic salt ions to form precipitates that release Ni^2+^ and inhibit crop uptake of Ni^2+^, thereby achieving the promotion of the encoded C-P lyase process that in turn enhances AP; (2) Zn^2+^ accumulation may selectively promote the colonization of microorganisms that can tolerate or utilize high Zn^2+^ levels [61], and if there are species of these microorganisms with high expression levels of *phnP* genes, the abundance of the proteins encoded by these genes will increase accordingly, and this in turn will increase AP. The C-P lyase pathway is required for organisms to recycle inorganic P from phosphonate; *phnP* gene expression is up-regulated when phosphate supply is limited, and some microorganisms use phosphonate compounds more efficiently to produce phosphate via enzymes that break stable C-P bonds, i.e., Zn^2+^ significantly facilitates the process of transitioning between phosphonate and hypophosphonate metabolism and pentose phosphate metabolism in the P cycle.

### 4.3. Implications

This study underscores the importance of considering dynamics within the microbial community and interactions with mineral ions when formulating soil management strategies aimed at enhancing the efficacy of P addition. Our findings emphasize the significant role of Zn^2^⁺ in modulating microbial interactions and P activation in agricultural soils through *gcd* gene-mediated phosphorus solubilization mechanisms. The dominance of Actinobacteria in our metagenomic datasets establishes this phylum as the key driver for implementing Zn^2+^-regulated P management strategies.

For red and yellow soils in the Anning River Basin, we recommend: (1) Zn^2+^ concentration regulation: Tailor Zn supplementation to local soil types, with 5–12 mg·kg ⁻ ¹ recommended for red/yellow soils based on trials in Yunnan showing 15–20% improved phosphorus activation. (2) Risk management: Adhere to WHO (200 mg·kg ⁻ ¹ Zn threshold) and EPA guidelines, establish a tiered warning system, and deploy GIS-guided variable-rate fertilization systems for precise 0–20 cm soil layer application. (3) Microbial formulation: Use a ratio of *Streptomyces* (phosphorus-solubilizing), *Bacillus megaterium* (siderophore-producing), and *Arthrobacter nicotianae* (Zn-tolerant) strains. Apply near-infrared-equipped fertilizer machines for 0.5 kg/mu minimum control, combining zinc side-dressing with foliar microbial sprays at maize nodulation stages to align with phosphorus demand peaks.

While zinc fertilizer supplementation increased costs, this may have been offset by phosphorus fertilizer as well as savings and increased yields from the biofungicides used. Since this study has not been validated with specific zinc fertilizer-biomycorrhizal applications, future small-scale experiments could be conducted to specifically measure economic costs based on the findings of this study. Meanwhile, future research needs to further validate the applicability of this mechanism in different agroecosystems in order to accelerate the translation of theoretical results into sustainable practices.

This theoretical innovation not only constructs the coupling framework of “mineral ion-microbial metabolism-nutrient cycling”, but also provides new targets for the development of targeted microbial preparations and cleaner production technologies, and promotes the transformation of agriculture to the recycling mode of “reduce-reuse-recycle” through the synergistic Zn-P management strategy. In addition, the synergistic management strategy of Zn-P can facilitate the transformation of agriculture into a “reduce-reuse-recycle” model.

## 5. Conclusions

Collectively, by integrating biogeochemical cycles, microbial ecology and agricultural engineering perspectives, this study reveals the central role of the coupled Zn^2+^-microbial-phosphorus cycle mechanism in agricultural soil systems, breaks through the traditional perception of Zn^2+^ as a chemical accelerator only, and elucidates its role as a microbial nutrient factor that regulates the life activities by interdisciplinary mechanisms that reshape the phosphorus cycle network: Zn^2+^ accumulation significantly affected the AEP hydrolytic degradation pathway in the P cycle, which is beneficial to phosphate production; Zn^2+^ accumulation promoted *phnP* in the C-P cleaving enzyme process in the P cycle. These findings provide novel insights into understanding soil P cycling mechanisms while also offering valuable perspectives for soil fertility management and improvement.

However, to fully realize the application potential of this theory, the following key scientific issues still need to be addressed: first, the long-term environmental effects of Zn^2+^ accumulation are still unclear, and the potential thresholds of its sustained inputs on the stability of soil microbial communities and phosphorus cycling functions need to be further assessed; second, the interaction mechanisms between Zn^2+^ and other micronutrients (e.g., Fe, Second, the interaction mechanism between Zn^2+^ and other micronutrients (e.g., Fe, Cu) needs to be elucidated, especially the effects of competitive uptake or synergistic effects on microbial metabolic networks. Finally, the crop species-specific response patterns need to be systematically analyzed, and the prediction model for the efficiency of Zn-phosphorus coupling of different species needs to be established to guide the precision agriculture practice. Meanwhile, future research should be based on the breakthrough of the above bottlenecks to carry out field-scale validation, so as to accelerate the transformation of the theoretical results into sustainable practice.

## Supporting information

S1 Table Comparison of TP and AP content in some regions of China. The average value of total phosphorus (TP) in farmland soils in the Anning River Basin was 0.73 g/kg; the average value of available phosphorus (AP) in farmland soils in the Anning River Basin was 0.07 g/kg.
(DOCX)

S2 Table Microbial phosphorus cycle related genes. (DOCX)

S3 Table Spearman correlation between mineral ions and phosphorus component. (DOCX)

S1 Fig TP and AP in farmland of China. Base map: Authorized by the Ministry of Natural Resources, China, Map Review Approval Number: GS(2019)1822.
(DOCX)

S2 Fig Spearman correlation heatmap. (DOCX)

S3 Fig Sankey diagram of phylum and class of microorganisms. a. Sankey diagram of phylum and class of microorganisms that carry *gcd*; b. Sankey diagram of phylum and class of microorganisms that carry *phnw*.
(DOCX)

S4 Fig a. Soil microphytobenthic species-level co-occurrence network, with nodes denoting individual species and node size denoting the species’ degree, coloured separately according to major ecological clusters; b. Population characteristics of dominant species in major ecological clusters at the phylum level. (DOCX)

S5 Fig Abundance of major microbial ecological clusters in soils of the Anning River Basin. (DOCX)

S6 Fig The linear relationships between the topological characteristics of the co-occurring networks of Module and soil microbiota and AP and Zn^2+^ were statistically analysed using ordinary least squares linear regression. (DOCX)

S7 Fig a. Co-linear network of species carrying the *phnW*; b Population characteristics of dominant species in major ecological clusters at the phylum level. (DOCX)

S8 Fig The Module abundance of spieces carrying *phnW.* (DOCX)

S9 Fig The Module abundance of spieces carrying *phnW* and AP and Zn^2+^ were statistically analysed using ordinary least squares linear regression. (DOCX)

S10 Fig Partial correlations analysis between mineral ions and phosphorus fractions. (DOCX)
