# Supplementary material for: Zinc ion increases the effectiveness of phosphorus in agricultural soils through microbial solubilization
Source: PLoS One. 2025 Dec 15;20(12):e0327961. doi: 10.1371/journal.pone.0327961 (PMC12704886; doi:10.1371/journal.pone.0327961)
Supplement: S1 Table — The average value of total phosphorus (TP) in farmland soils in the Anning River Basin was 0.73 g/kg; the average value of available phosphorus (AP) in farmland soils in the Anning River Basin was 0.07 g/kg. (DOCX) [file pone.0327961.s001.docx]

**S1 Table. Comparison of TP and AP content in some regions of China.** The average value of total phosphorus (TP) in farmland soils in the Anning River Basin was 0.73 g/kg; the average value of available phosphorus (AP) in farmland soils in the Anning River Basin was 0.07 g/kg.

| **Region** | **TP(g/kg)** | **AP(g/kg)** |
| --- | --- | --- |
| Beijing | 0.800 | 0.050 |
| Shanghai | 0.750 | 0.045 |
| Guangdong | 0.820 | 0.055 |
| Sichuan | 0.790 | 0.050 |
| Henan | 0.810 | 0.052 |
| Shandong | 0.830 | 0.057 |
| Jiangsu | 0.805 | 0.053 |
| Zhejiang | 0.795 | 0.050 |
| Hubei | 0.820 | 0.055 |
| Hubnan | 0.800 | 0.052 |
| Anning River Basin | 0.737 | 0.070 |
