# Supplementary material for: Zinc ion increases the effectiveness of phosphorus in agricultural soils through microbial solubilization
Source: PLoS One. 2025 Dec 15;20(12):e0327961. doi: 10.1371/journal.pone.0327961 (PMC12704886; doi:10.1371/journal.pone.0327961)
Supplement: S2 Table — (DOCX) [file pone.0327961.s002.docx]

**S2 Table. Microbial phosphorus cycle related genes.**

| **Microbial phosphorus cycle related genes** | | | |
| --- | --- | --- | --- |
| **Functional gene grouping** | **Gene Function Detials** | **Gene** | **KO** |
| Solubilization | Quinoprotein glucose dehydrogenase | *gcd* | K00117 |
|  | Glutaconyl-CoA decarboxylase subunit alpha | *gcdA* | K01615 |
|  | Carboxybiotin decarboxylase | *gcdB* | K20509 |
|  | Glutaconyl-CoA/methylmalonyl-CoA decarboxylase subunit gamma | *gcdC* | K23351 |
|  | Glutaconyl-CoA/methylmalonyl-CoA decarboxylase subunit delta | *gcdD* | K23352 |
|  | Inorganic pyrophosphatase | *ppa* | K01507 |
|  | Manganese-dependent inorganic pyrophosphatase | *ppaC* | K15986 |
|  | Pyrophosphatase PpaX | *ppaX* | K06019 |
|  | Serine/Threonine-protein kinase PpkA | *ppkA* | K11912 |
|  | Polyphosphate kinase | *ppk1* | K00937 |
|  | Polyphosphate kinase | *ppk2* | K22468 |
|  | Guanosine-5'-triphosphate,3'-diphosphate pyrophosphatase | *ppx* | K01524 |
| Mineralization | 4-phytase / Acid phosphatase | *appA* | K01093 |
|  | Glycerol transport system permease protein | *glpQ* | K17323 |
|  | Proteasome accessory factor A | *pafA* | K13571 |
|  | Alkylphosphonate utilization operon protein | *phnA* | K06193 |
|  | Phosphonate transport system regulatory protein | *phnF* | K02043 |
|  | C-P lyase multienzyme complex | *phnG* | K06166 |
|  | C-P lyase multienzyme complex | *phnH* | K06165 |
|  | C-P lyase multienzyme complex | *phnI* | K06164 |
|  | C-P lyase multienzyme complex | *phnJ* | K06163 |
|  | C-P lyase multienzyme complex | *phnL* | K05780 |
|  | C-P lyase multienzyme complex | *phnM* | K06162 |
|  | C-P lyase multienzyme complex | *phnN* | K05774 |
|  | Phosphoribosyl 1,2 -cyclic phosphate phosphodiesterase | *phnP* | K06167 |
|  | 2aminoethylphosphonate-pyruvate transaminase | *phnW* | K03430 |
|  | Phosphonoacetaldehyde hydrolase | *phnX* | K05306 |
|  | 2-aminoethylphosphonate dioxygenase | *phnY* | K21195 |
|  | Acid phosphatase (Fungi) | *phoA* | K01078 |
|  | Alkaline phosphatase D | *phoD* | K01113 |
|  | Acid phosphatase (class A) | *phoN* | K09474 |
|  | Phloretin hydrolase | *phy* | K22906 |
|  | Glycerophosphoryl diester phosphodiesterase | *ugpQ* | K01126 |
|  | Phosphotriesterase-related protein | *php* | K07048 |
|  | Glycerol-3-phosphate dehydrogenase | *glpA,glpD* | K00111 |
|  | Glycerol-3-phosphate dehydrogenase subunit B | *glpB* | K00112 |
|  | Glycerol-3-phosphate dehydrogenase subunit C | *glpC* | K00113 |
|  | Glycerol uptake facilitator protein | *glpF* | K02440 |
|  | Glycerol kinase | *glpK* | K00864 |
|  | Glycerol-3-phosphate regulon repressor | *glpR* | K02444 |
|  | Phosphoribosyl 1,2-cyclic phosphate 1,2-diphosphodiesterase | *phnPP* | K20859 |
| Regulation | Alkaline phosphatase | *phoB* | K01077 |
|  | Phosphate regulon sensor histidine kinase | *phoR* | K07636 |
|  | Negative regulator of PhoR/PhoB two-component regulator | *phoU* | K02039 |
|  | Two-component system, OmpR family, response regulator PhoP | *phoP* | K07660 |
|  | Two-component system, OmpR family, alkaline phosphatase synthesis response regulator PhoP | *phoB1,phoP* | K07658 |
|  | Two-component system, OmpR family, response regulator RegX3 | *regX3* | K07776 |
|  | Two-component system, OmpR family, sensor histidine kinase SenX3 | *senX3* | K07768 |
| Transportation | Inorganic phosphate transporter | *pit* | K03306 |
|  | Phosphate transport system permease protein | *pstA* | K02038 |
|  | Phosphate transport system ATP-binding protein | *pstB* | K02036 |
|  | Phosphate transport system permease protein | *pstC* | K02037 |
|  | Phosphate transport system substrate-binding protein | *pstS* | K02040 |
|  | Phosphonate transport system ATP-binding protein | *phnC* | K02041 |
|  | Phosphonate transport system substrate-binding protein | *phnD* | K02044 |
|  | Phosphonate transport system permease protein | *phnE* | K02042 |
|  | 2-aminoethylphosphonate transport system substrate-binding protein | *phnS* | K11081 |
|  | 2-aminoethylphosphonate transport system ATP-binding protein | *phnT* | K11084 |
|  | 2-aminoethylphosphonate transport system permease protein | *phnU* | K11083 |
|  | 2-amino-ethyl phosphonate transport system permease protein | *phnV* | K11082 |
|  | sn-glycerol 3-phosphate transport system permease protein | *ugpA* | K05814 |
|  | sn-glycerol 3-phosphate transport system substrate-binding protein | *ugpB* | K05813 |
|  | sn-glycerol 3-phosphate transport system ATP-binding protein | *ugpC* | K05816 |
|  | sn-glycerol 3-phosphate transport system permease protein | *ugpE* | K05815 |
