# Supplementary material for: Zinc ion increases the effectiveness of phosphorus in agricultural soils through microbial solubilization
Source: PLoS One. 2025 Dec 15;20(12):e0327961. doi: 10.1371/journal.pone.0327961 (PMC12704886; doi:10.1371/journal.pone.0327961)
Supplement: S3 Table — (DOCX) [file pone.0327961.s003.docx]

**S3 Table. Spearman correlation between mineral ions and phosphorus component.**

| **Spearman correlation** | | | | |
| --- | --- | --- | --- | --- |
|  | AP | Pi | Po | TP |
| Mg^2+^ | -0.28 | 0.22 | -0.032 | 0.005 |
| Zn^2+^ | 0.755** | 0.24 | 0.499** | 0.465** |
| Ca^2+^ | 0.366* | 0.371* | 0.623** | 0.629** |
| Mn^2+^ | 0.605** | 0.124 | 0.324* | 0.291 |
| Al^3+^ | 0.204 | -0.037 | 0.092 | 0.061 |
| Cu^2+^ | -0.131 | 0.134 | -0.135 | -0.114 |
| WS-Fe | -0.063 | -0.135 | -0.122 | -0.145 |
| WS-Si | 0.005 | 0.12 | 0.078 | 0.081 |
| * P < 0.05 ** P < 0.01 | | |  |  |
