# Supplementary material for: Zinc ion increases the effectiveness of phosphorus in agricultural soils through microbial solubilization
Source: PLoS One. 2025 Dec 15;20(12):e0327961. doi: 10.1371/journal.pone.0327961 (PMC12704886; doi:10.1371/journal.pone.0327961)
Supplement: S1 Fig — Base map: Authorized by the Ministry of Natural Resources, China, Map Review Approval Number: GS(2019)1822. (DOCX) [file pone.0327961.s004.docx]

**S1 Fig. TP and AP in farmland of China**

Base map: Authorized by the Ministry of Natural Resources, China, Map Review Approval Number : GS(2019)1822.

**
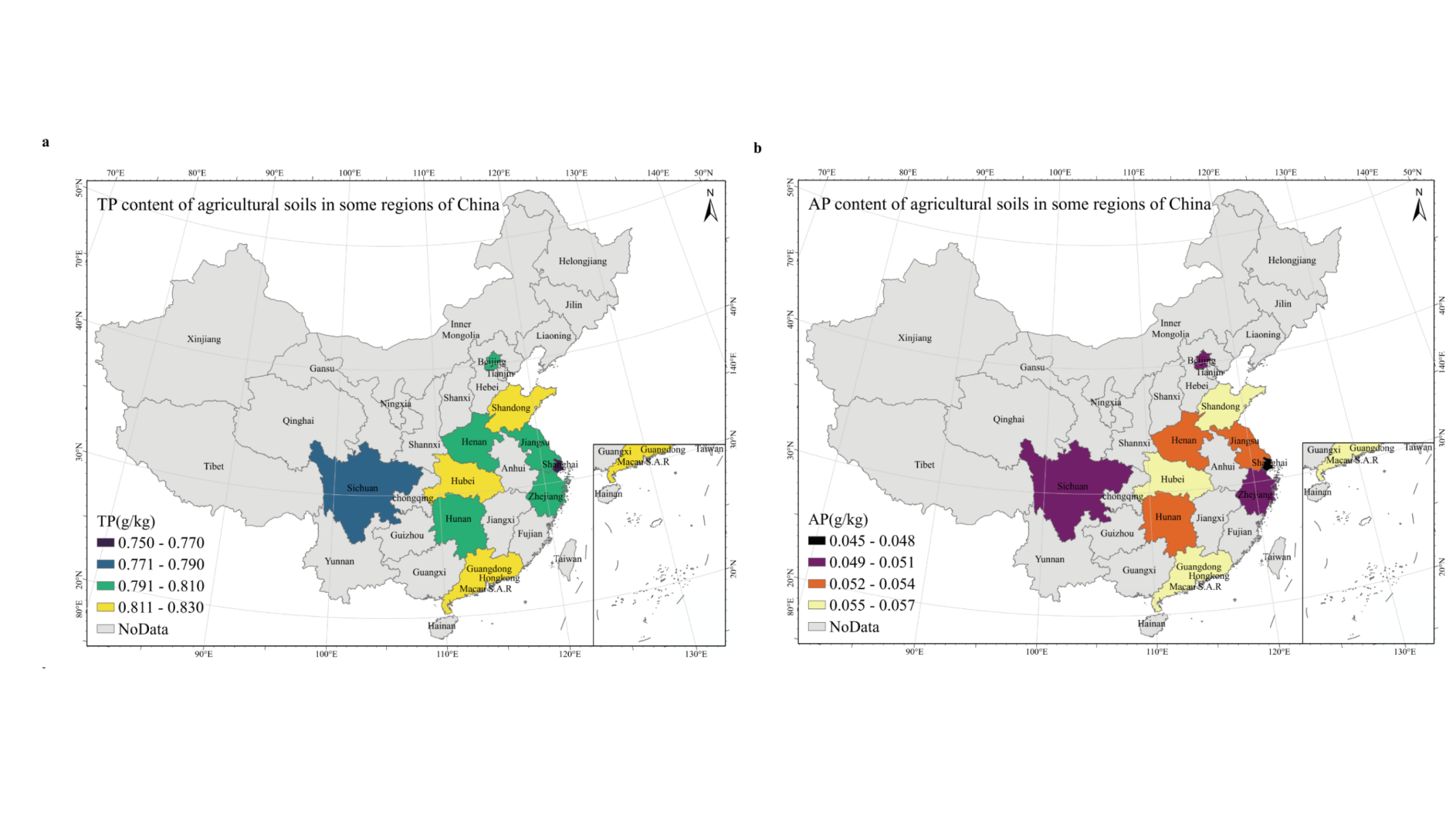
**
