# Supplementary figures and images for: Zinc ion increases the effectiveness of phosphorus in agricultural soils through microbial solubilization
Source: PLoS One. 2025 Dec 15;20(12):e0327961. doi: 10.1371/journal.pone.0327961 (PMC12704886; doi:10.1371/journal.pone.0327961)

**S2 Fig. Spearman correlation heatmap.**


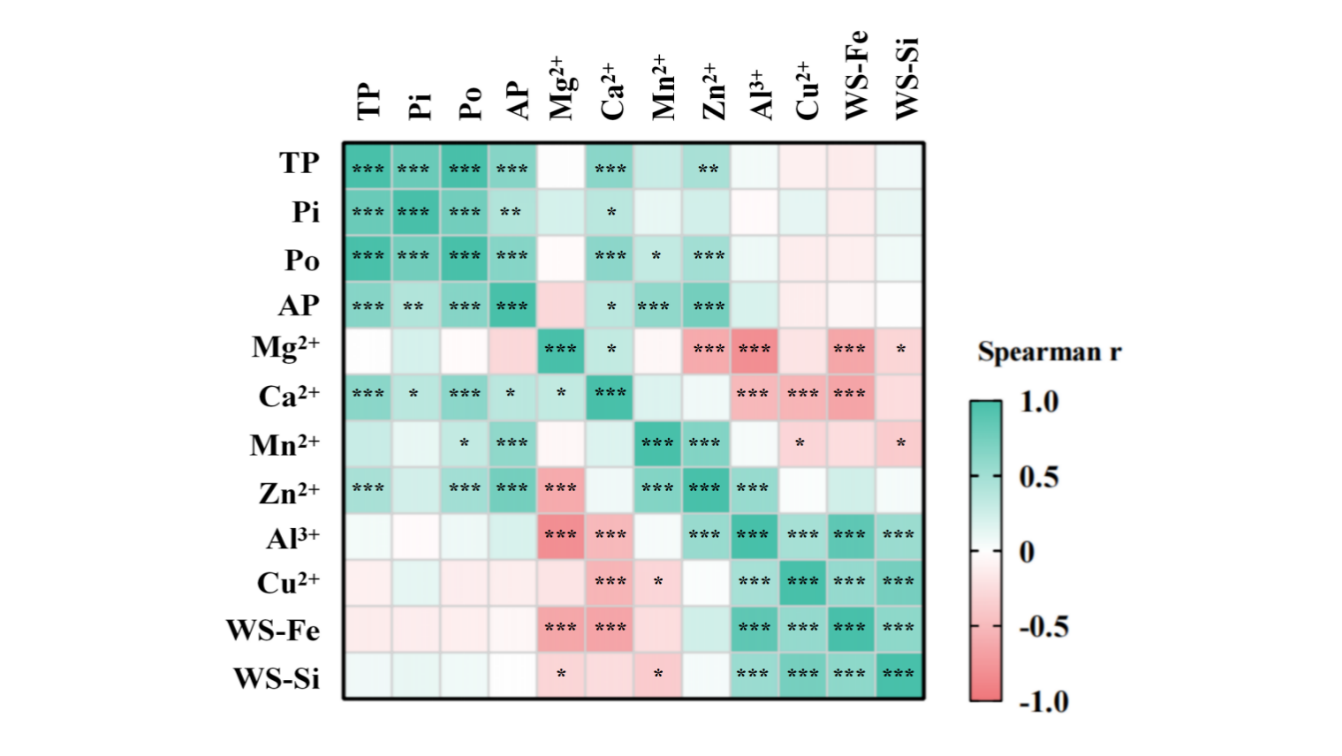

Supplement: S2 Fig — (DOCX) [file pone.0327961.s005.docx]

**S5 Fig. Abundance of major microbial ecological clusters in soils of the Anning River Basin.**


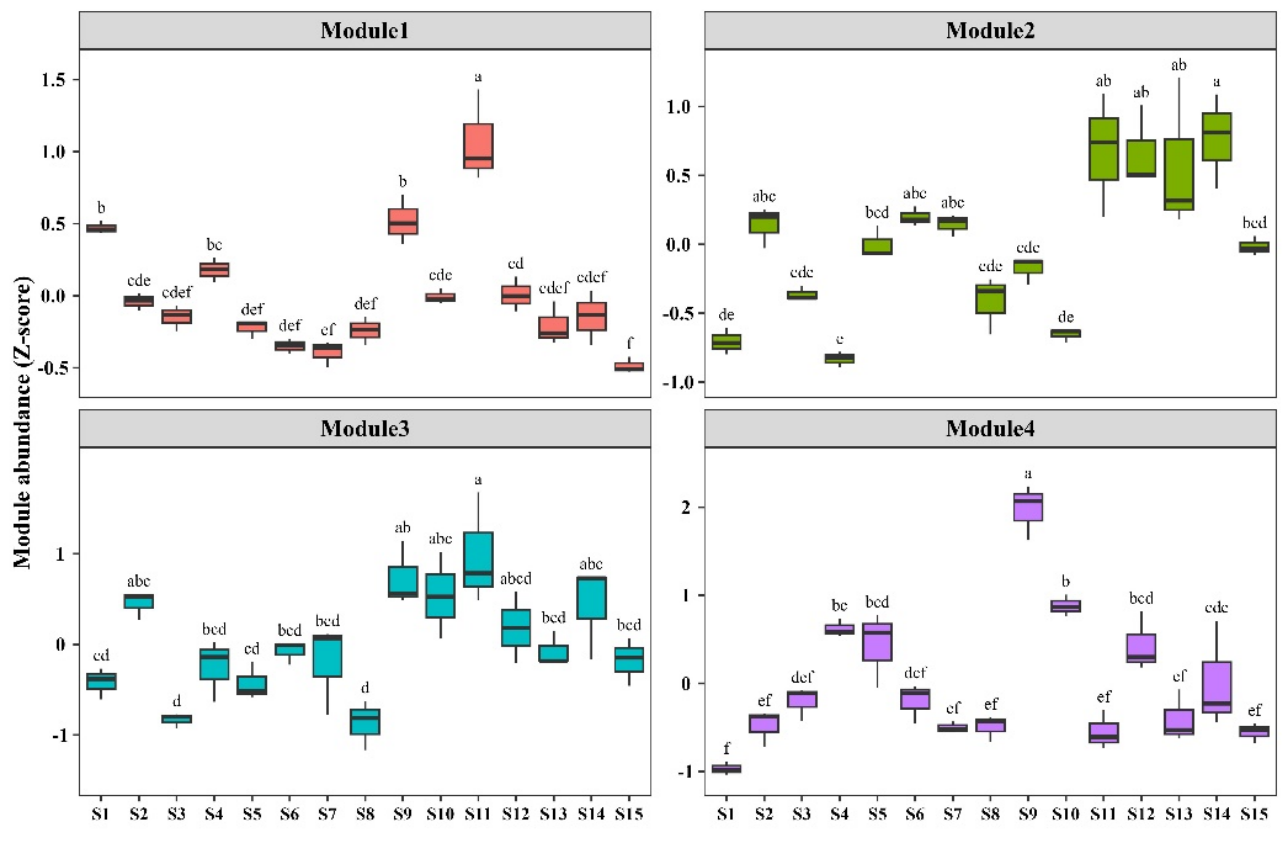

Supplement: S5 Fig — (DOCX) [file pone.0327961.s008.docx]

**S8 Fig. The Module abundance of spieces carrying *phnW*.**


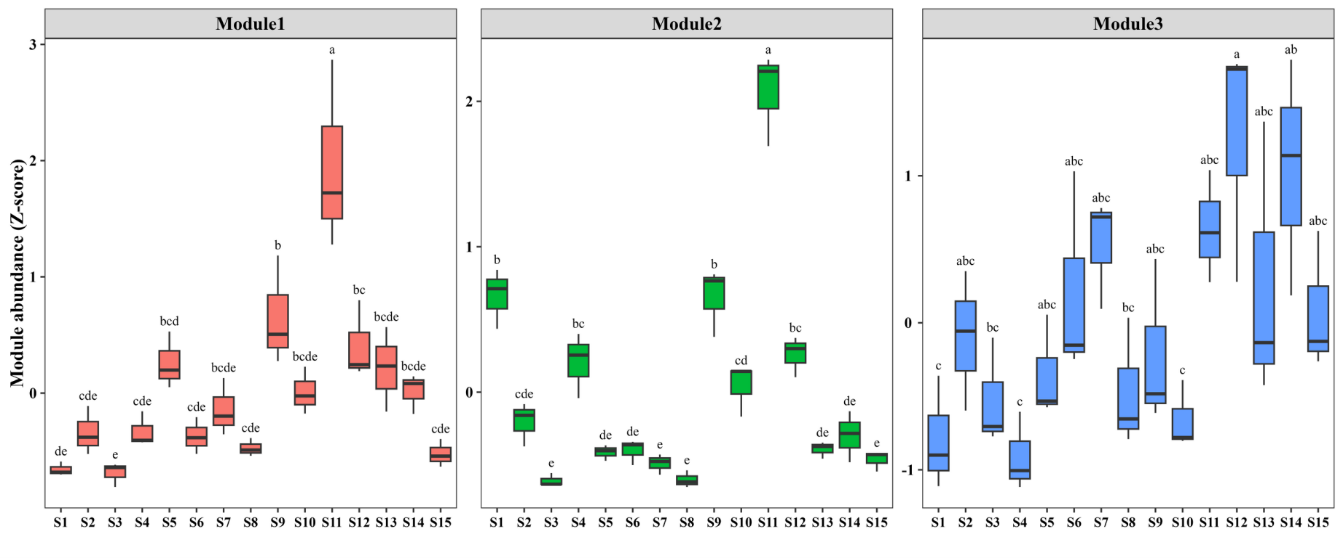

Supplement: S8 Fig — (DOCX) [file pone.0327961.s011.docx]

**S10 Fig. Partial correlations analysis between mineral ions and phosphorus fractions.**


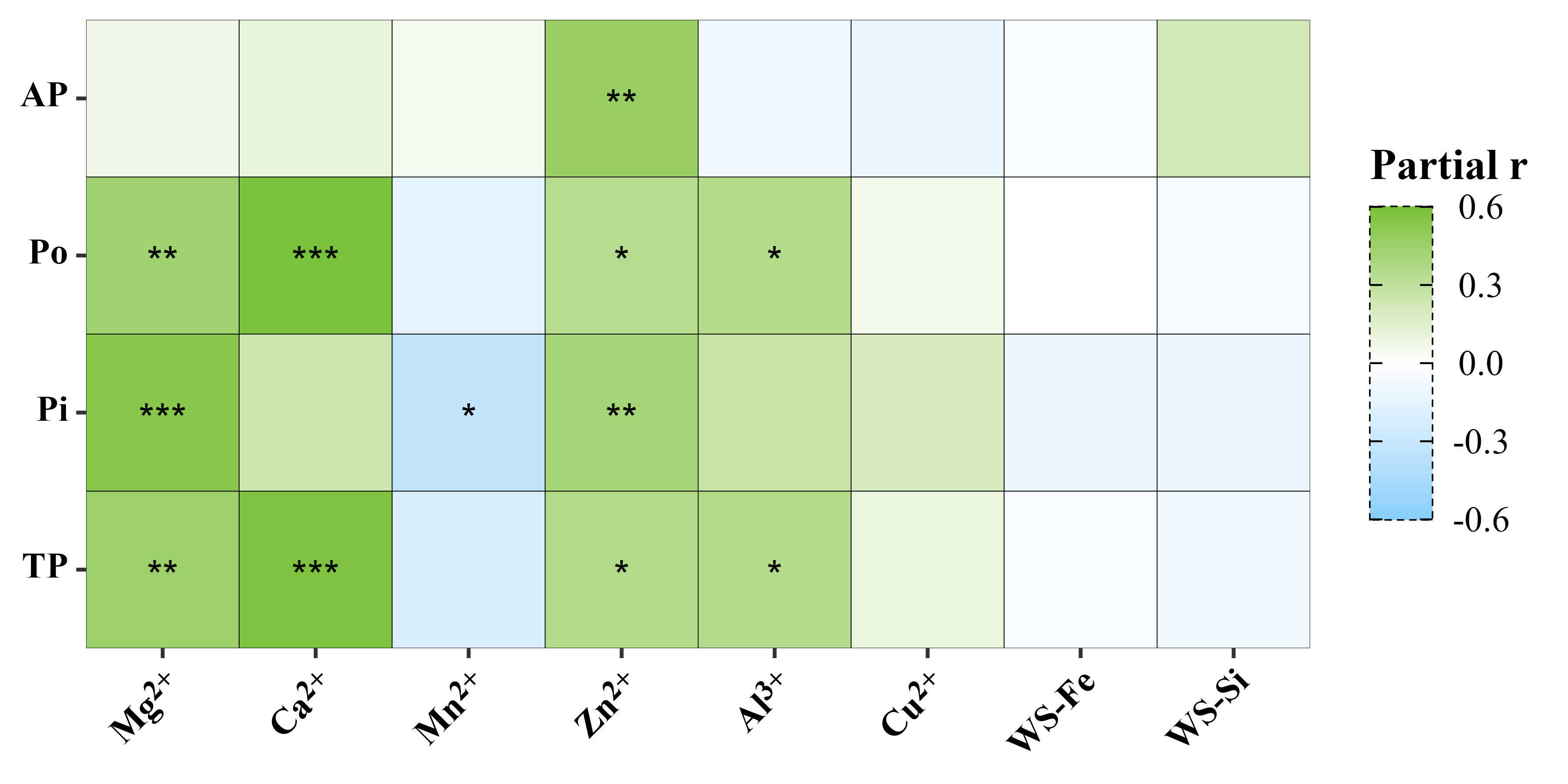

Supplement: S10 Fig — (DOCX) [file pone.0327961.s013.docx]
