# Supplementary material for: Zinc ion increases the effectiveness of phosphorus in agricultural soils through microbial solubilization
Source: PLoS One. 2025 Dec 15;20(12):e0327961. doi: 10.1371/journal.pone.0327961 (PMC12704886; doi:10.1371/journal.pone.0327961)

**S3 Fig. Sankey diagram of phylum and class of microorganisms.** a. Sankey diagram of phylum and class of microorganisms that carry *gcd*; b. Sankey diagram of phylum and class of microorganisms that carry *phnw*.


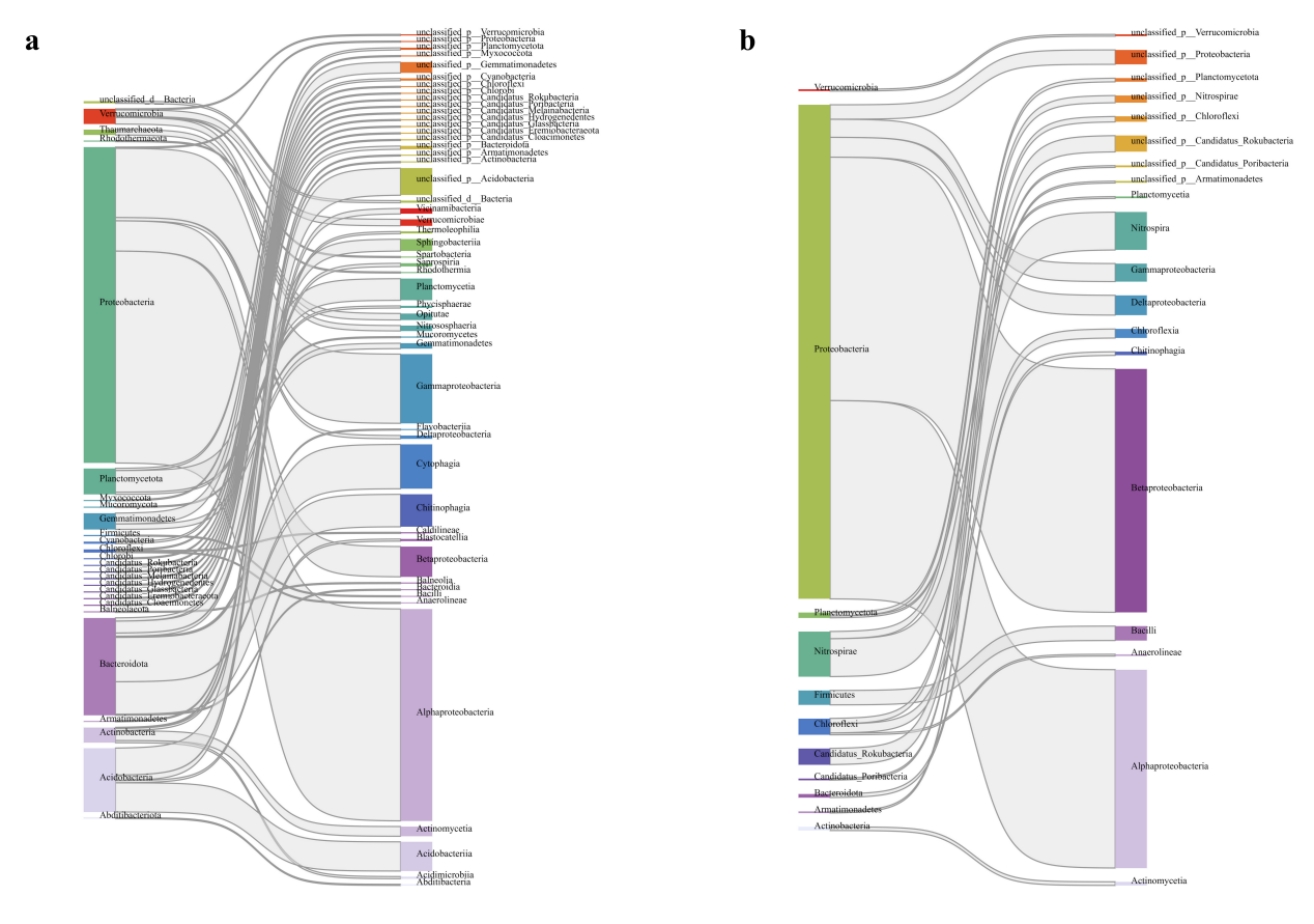

Supplement: S3 Fig — a. Sankey diagram of phylum and class of microorganisms that carry gcd; b. Sankey diagram of phylum and class of microorganisms that carry phnw. (DOCX) [file pone.0327961.s006.docx]
