# Supplementary material for: Zinc ion increases the effectiveness of phosphorus in agricultural soils through microbial solubilization
Source: PLoS One. 2025 Dec 15;20(12):e0327961. doi: 10.1371/journal.pone.0327961 (PMC12704886; doi:10.1371/journal.pone.0327961)

**S4 Fig. a. Soil microphytobenthic species-level co-occurrence network**, **with nodes denoting individual species and node size denoting the species' degree, coloured separately according to major ecological clusters; b. Population characteristics of dominant species in major ecological clusters at the phylum level.**


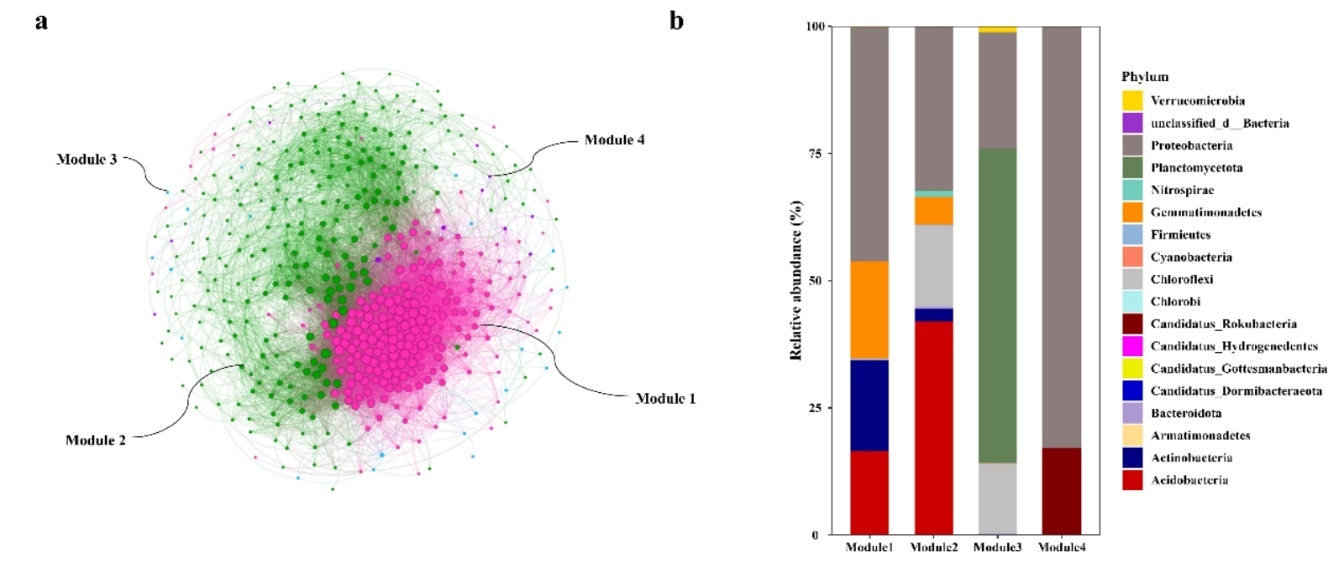

Supplement: S4 Fig — (DOCX) [file pone.0327961.s007.docx]
