# Supplementary material for: Zinc ion increases the effectiveness of phosphorus in agricultural soils through microbial solubilization
Source: PLoS One. 2025 Dec 15;20(12):e0327961. doi: 10.1371/journal.pone.0327961 (PMC12704886; doi:10.1371/journal.pone.0327961)

**S6 Fig. The linear relationships between the topological characteristics of the co-occurring networks of Module and soil microbiota and AP and Zn^2+^ were statistically analysed using ordinary least squares linear regression.**


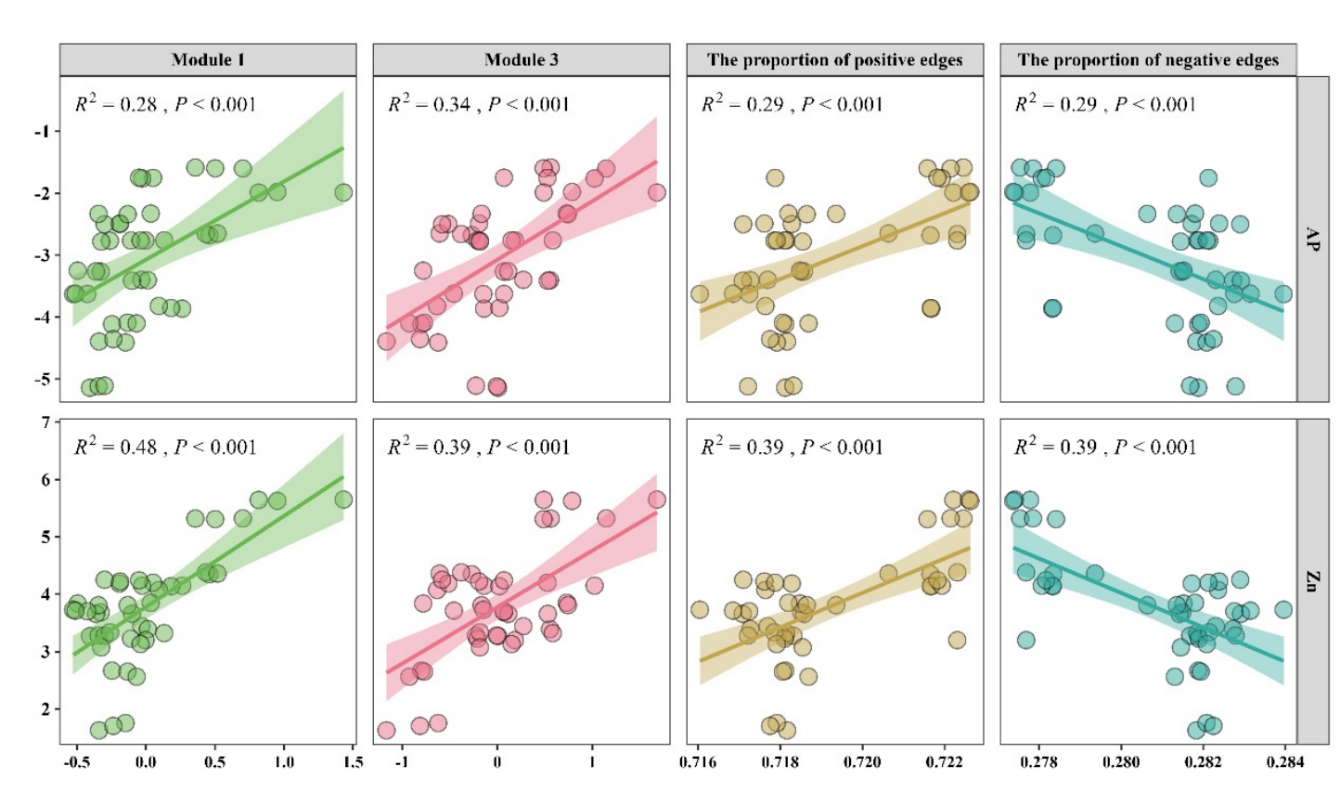

Supplement: S6 Fig — (DOCX) [file pone.0327961.s009.docx]
