# Supplementary material for: Zinc ion increases the effectiveness of phosphorus in agricultural soils through microbial solubilization
Source: PLoS One. 2025 Dec 15;20(12):e0327961. doi: 10.1371/journal.pone.0327961 (PMC12704886; doi:10.1371/journal.pone.0327961)

**S7 Fig. a. Co-linear network of species carrying the *phnW*; b Population characteristics of dominant species in major ecological clusters at the phylum level.**


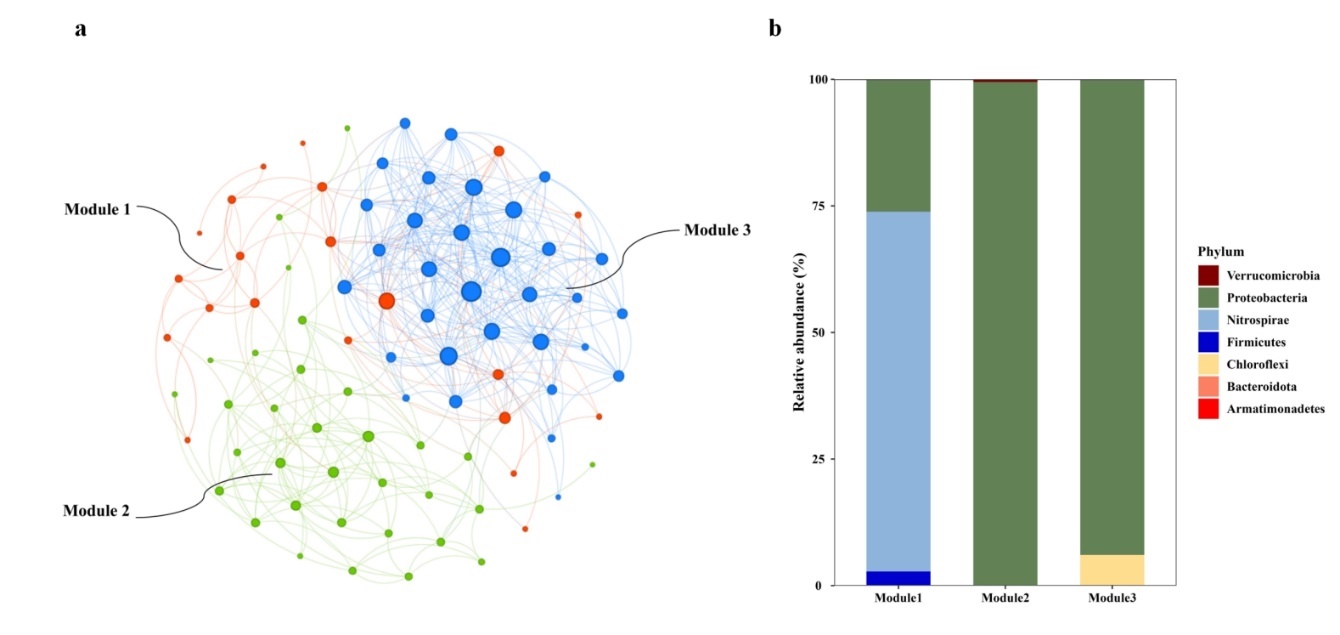

Supplement: S7 Fig — (DOCX) [file pone.0327961.s010.docx]
