# Supplementary material for: Zinc ion increases the effectiveness of phosphorus in agricultural soils through microbial solubilization
Source: PLoS One. 2025 Dec 15;20(12):e0327961. doi: 10.1371/journal.pone.0327961 (PMC12704886; doi:10.1371/journal.pone.0327961)

**S9 Fig. The Module abundance of spieces carrying *phnW* and AP and Zn^2+^ were statistically analysed using ordinary least squares linear regression.**


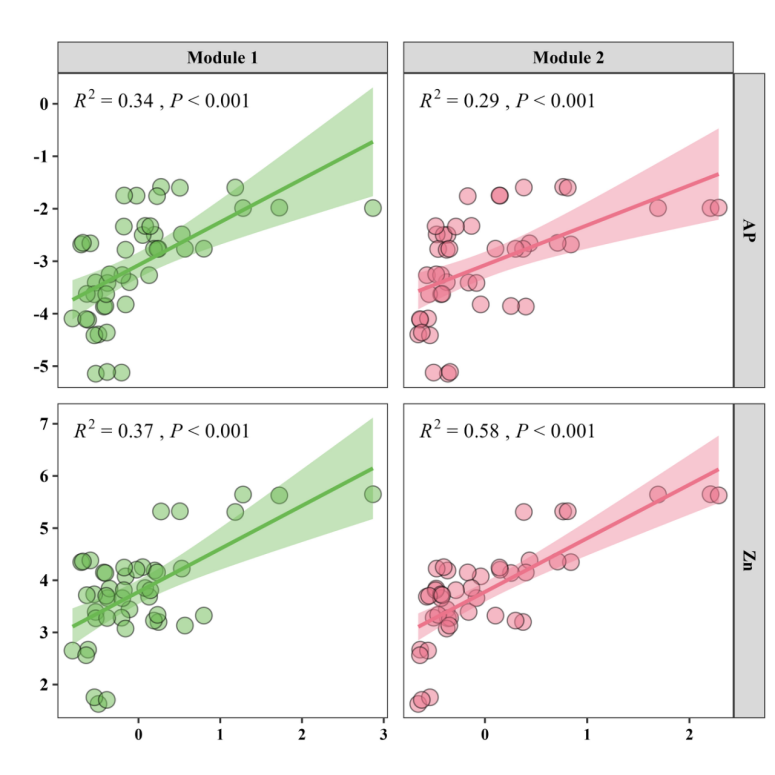

Supplement: S9 Fig — (DOCX) [file pone.0327961.s012.docx]
